# Next-Generation Sequencing Analysis of Mutations in Circulating Tumor DNA from the Plasma of Patients with Head–Neck Cancer Undergoing Chemo-Radiotherapy Using a Pan-Cancer Cell-Free Assay

**DOI:** 10.3390/curroncol30100643

**Published:** 2023-09-29

**Authors:** Michael I. Koukourakis, Erasmia Xanthopoulou, Ioannis M. Koukourakis, Sotirios P. Fortis, Nikolaos Kesesidis, Christos Kakouratos, Ioannis Karakasiliotis, Constantin N. Baxevanis

**Affiliations:** 1 Department of Radiotherapy—Oncology, Medical School, Democritus University of Thrace, 68100 Alexandroupolis, Greece; mia_x1995@hotmail.com (E.X.); ckakouratos@gmail.com (C.K.); 2 Radiation Oncology Unit, 1st Department of Radiology, Aretaieion University Hospital, 11528 Athens, Greece; koukourioannis@gmail.com; 3 Cancer Immunology and Immunotherapy Center, Cancer Research Center, Saint Savas Cancer Hospital, 11522 Athens, Greece; sfortis1989@gmail.com (S.P.F.); costas.baxevanis@gmail.com (C.N.B.); 4 Laboratory of Biology, Medical School, Democritus University of Thrace, 68100 Alexandroupolis, Greece; nkesesid@med.duth.gr (N.K.); ioakarak@med.duth.gr (I.K.)

**Keywords:** head neck cancer, radiotherapy, ctDNA, NGS, TP53, gene mutations

## Abstract

Using next-generation sequencing (NGS), we investigated DNA mutations in the plasma tumor cell-free circulating DNA (ctDNA) of 38 patients with inoperable squamous cell head neck cancer (SCHNC) before and after the completion of chemoradiotherapy (CRT). Baseline mutations of the *TP53* were recorded in 10/38 (26.3%) and persisted in 4/10 patients after CRT. *ΤP53* mutations were further detected post CRT in 7/38 additional patients with undetectable mutations at baseline (overall rate 44.7%). Furthermore, 4/38 patients exhibited baseline mutations of the *EGFR*, *AR*, *FGFR3*, and *FBXW3,* and four new gene mutations were detected after CRT (*MTOR*, *EGFR3*, *ALK*, and *SF3B1)*. Τ4 stage was related with a significantly higher rate of mutations (*TP53* and overall). Mutations were observed in 8/30 (26.6%) responders (complete/partial response) vs. in 6/8 (75%) of the rest of the patients (*p* = 0.03). Significant poorer LRFS was noted for patients with mutations detected before and after CRT (*p* = 0.02). Patients who had detectable mutations either before or after CRT had significantly worse DMFS (*p* = 0.04 overall, and *p* = 0.02 for *TP53* mutations). It was concluded that assessment of mutations before and after the end of CRT is essential to characterize patients with a high risk of locoregional recurrence or metastatic progression.

## 1. Introduction

During disease progression, cancer cells and especially stem cells acquire genetic mutations that define clinical aggressiveness, invasion, metastasis, and resistance to radiotherapy (RT) and chemotherapy [1,2]. Such mutations can appear even during therapy, either as a result of direct DNA damage and failure to properly repair the DNA strand breaks or as an accumulation and prevalence of existing resistant cancer cell clones with specific mutations [3,4].

The profile of genetic mutations of a tumor can be assessed with next-generation sequencing (NGS) based on tissue biopsy material. Indeed, this has been established as a routine test to identify molecular fingerprints that can guide therapy with molecular inhibitors or monoclonal antibodies, e.g., therapies targeting epidermal growth factor receptor (EGFR) or other gene mutations [5]. Nevertheless, NGS can also be applied in cell-free DNA (cfDNA) extracted from the plasma or the saliva and other body fluids of patients. Fragmented DNA released by cancer cells through vesicles and exosomes or fragments from dying cancer cells that enter the circulation (circulating tumor DNA-ctDNA) can be isolated from the blood and body fluids. Testing ctDNA for tumor mutations in liquid biopsies has emerged as a convenient and reliable method for tumor profiling. In fact, Parkh et al. suggested that analysis of a single-lesion tumor biopsy alone is less effective than ctDNA analysis in identifying tumor genetic heterogeneity and alterations associated with resistance to therapy [6]. Liquid biopsies, being non-invasive procedures, can be repeatedly obtained from patients without any discomfort during their therapy, conferring an important advantage over tissue biopsy analysis.

Squamous cell head neck cancer (SCHNC) accounts for approximately 600,000 new cases annually, ranking 7th in prevalence among different cancer subtypes, with smoking and human papilloma virus (HPV) infections being major risk factors [7]. Combination of surgery and adjuvant RT or chemoradiotherapy (CRT) and definitive/radical RT or CRT for inoperable cases are the established treatment modalities of this malignancy, offering high curability rates. While the incidence of distant metastases is less than 30%, locoregional recurrence eventually occurs in more than 50% of patients with a locally advanced disease [8]. Specifically, the 2-year progression-free survival rates of locally advanced head and neck cancer patients treated with radical CRT range from 30% to 60% [9].

A number of studies investigating the cfDNA levels in the plasma or other body fluids of SCHNC patients before or after administration of RT or CRT have suggested that this method could potentially predict response to treatment and patient prognosis [10]. In a prospective trial, we quantitatively assessed the cfDNA concentration in the plasma of a cohort of patients with locally advanced SCHNC treated with CRT [11]. Increased levels were noted in 55% of patients and this was related to poorer response to therapy and worse prognosis. Beyond the quantity of cfDNA, gene mutation analysis could identify ctDNA that would ultimately prove to be of further prognostic and predictive relevance. In the current study, we report the analysis of DNA mutations in this cohort of patients. Detection of mutations was based on a panel of selected genes involved in the cell cycle, cell death pathways, cell signaling, and metabolism. These were assessed before and at the end of CRT, aiming to identify specific gene mutations involved in resistance to CRT and also assess an eventual prognostic role of persistent or newly emerging mutations after treatment.

## 2. Materials and Methods

### 2.1. Patients 

As previously reported [11], patients with histologically diagnosed inoperable SCHNC were prospectively treated with RT combined with chemotherapy (cisplatin and/or cetuximab). Only patients with a conventional type of squamous cell cancer of the head–neck area, as identified by the 5th edition of the World Health Organization Classification of Head and Neck tumors [12], were included. Thirty-eight patients were included in the current analysis. No patient selection was performed (patients sequential in time). Inclusion criteria were good performance status (0–1), no previous chemotherapy or RT treatment, normal blood and biochemical tests, and absence of major heart, kidney, lung, autoimmune, hematological or psychiatric disease. Pregnant women were also excluded. Supplemental Table S1 presents details regarding patient and disease characteristics. The median follow-up was 15 months (2–36 months), while for patients alive at the time of last follow-up, this was 18 months (6–36 months).

### 2.2. Treatment Technique

Patients were treated with image-guided RT (IGRT) and a Volumetric Modulated Arc Therapy (VMAT) technique as previously reported [11]. Briefly, a simultaneous integrated boost (SIB) technique was applied to deliver 22 fractions, 5 fractions per week, within 30 days. Areas receiving prophylactic irradiation (e.g., neck) were treated with 2.15 Gy/fraction, while a daily booster dose of 0.40–0.55 Gy was administered to the primary tumor. The dose to enlarged nodes was increased using a daily booster dose of 0.3–0.4 Gy per fraction.

The SIB regimen has been widely applied in our department for the treatment of SCHNC, as this provides an equivalent dose delivered in 2 Gy fractions (EQD2) of 62–66 Gy, using the linear quadratic formula for tumor α/β = 4–10 Gy. As this dose is delivered with a 15-day acceleration, the time-corrected (T) EQD2-T (for a λ-value = 0.4–0.8 Gy/day) reaches an estimated biological dose of 68–78 Gy. This has been analyzed in detail in a previously reported study [11]. A recent radiobiological study by Shuryak et al. has suggested that optimized hypofractionated and accelerated RT in the range of the above-reported regimen can be better tolerated and is highly effective [13].

Patients received concurrent chemotherapy with intravenous administration of cisplatin at a dose of 35–40 mg/m^2^ per week, or cetuximab at a dose of 250 mg/m^2^/week, or a combination of both, as previously reported [14]. Thirteen patients were treated with cisplatin, four patients received cetuximab and twenty-one patients were treated with both agents.

### 2.3. Assessment of Response

A CT or an MRI scan was performed two months after RT completion to assess tumor response, and these were repeated six-monthly after that during the follow-up of patients. The WHO criteria [15] were applied to assess response to CRT as follows: complete response (CR) was defined as a 95–100% reduction in 2D dimensions of all detectable and measurable lesions. Partial (PR) and minimal response (MR) refer to 50–95% and 25–49% reduction in tumor dimensions (2D), respectively. An increase in tumor dimensions by more than 25% was defined as progressive disease. All other cases were considered to correspond to stable disease.

### 2.4. Plasma Collection

Twelve ml of venous blood were collected in vacuum blood collection test tubes containing ethylenediaminetetraacetic acid (EDTA) vials. The first sampling was performed immediately before the administration of the first RT fraction and chemotherapy infusion. A second blood sample was obtained on the day of the last RT fraction. The technique of PBMC and plasma isolation and storage has been previously reported [11].

### 2.5. Extraction and Quantification of Plasma cfDNA

Isolation of cell-free DNA was performed using the bead-based MagMAX™ Cell-free DNA extraction kit (catalog no.: A36716, Thermo Fisher Scientific, Waltham, MA, USA), specialized for high-quality isolation and specific enrichment of nucleic acids from liquid biopsies, as previously reported [11].

### 2.6. NGS Analysis

NGS analysis was performed using the Oncomine Pan-cancer cell-free assay (Thermo Fisher Scientific, USA; https://www.thermofisher.com/order/catalog/product/A37664, accessed on 25 September 2023) according to the manufacturer’s instructions. Library preparation was performed using cfDNA with concentrations ranging from 0.5 ng to 4 ng. Quantification of the isolated libraries was again performed via the Qubit system. The input range of each library used ranged from 10 ng to 20 ng. Sample analysis was performed using the Ion 540™ Kit-Chef system (Thermo Fisher Scientific). Automated preparation of Ion 540™ chips was performed, with each chip having six patient samples with different barcodes, and finally, sequencing of the samples was performed through the next-generation sequencing system using the Ion S5™ system. High-depth sequencing of the samples was performed using the Ion S5 sequencer, while the analysis of the samples was performed using Torrent Suite™ Software v. 5.12.3 and Ion Reporter™ version 5.20.2 (Thermo Fisher Scientific), using the Homo sapiens reference genome (hg19) as a reference library, according to the manufacturer’s instructions, for the analysis of point mutations, deletions, insertions, fusions and CNV’s in a panel of genes occurring in various cancer types, including *AKT1, ALK, AR, ARAF, BRAF, CHEK2, CTNNB1, DDR2, EGFR, ERBB2, ERBB3, ESR1, FGFR1, FGFR2, FGFR3, FGFR4, FLT3, GNA11, GNAQ, GNAS, HRAS, IDH1, IDH2, KIT, KRAS, MAP2K1, MAP2K2, MET, MTOR, NRAS, NTRK1, NTRK3, PDGFRA, PIK3CA, RAF1, RET, ROS1, SF3B1, SMAD4, SMO (*Hotspot genes (SNVs) and short indels*), ALK, BRAF, ERG, ETV1, FGFR1, FGFR2, FGFR3, MET, NTRK1, NTRK3, RET, ROS1 (Gene fusions), MET (*exon 14 skipping*), CCND1, CCND2, CCND3, CDK4, CDK6, EGFR, ERBB2, FGFR1, FGFR2, FGFR3, MET, MYC (CNVs) APC, FBXW7, PTEN, TP53* (Tumor suppressor genes), with a limit of detection (LOD) of a 0.1% allele frequency for SNVs and 1% for fusions. These genes are frequently mutated in multiple cancer types [16,17], including head and neck cancer [18].

### 2.7. Statistical Analysis

We used the GraphPad Prism 7.0 package for statistical analysis and graph presentation. The chi-square and Fisher’s exact t-test were used to test associations between categorical variables, as appropriate. Kaplan–Meier locoregional relapse-free survival (LRFS), disease-specific overall survival (OS), and distant metastasis-free survival (DMFS) curves were plotted. For statistical significance, we considered a *p*-value < 0.05.

## 3. Results

### 3.1. Gene Mutations

The quality control of the analyzed DNA samples from patients before and after CRT showed a QC-test-limit-of-detection LoD % range from 0.1 to 1.3 (median 0.4) and from 0.1 to 1.2 (median 0.5), respectively. Table 1 reports the genes, mutations, and molecular frequencies recorded in patients before and after CRT. 

**Figure 1:**
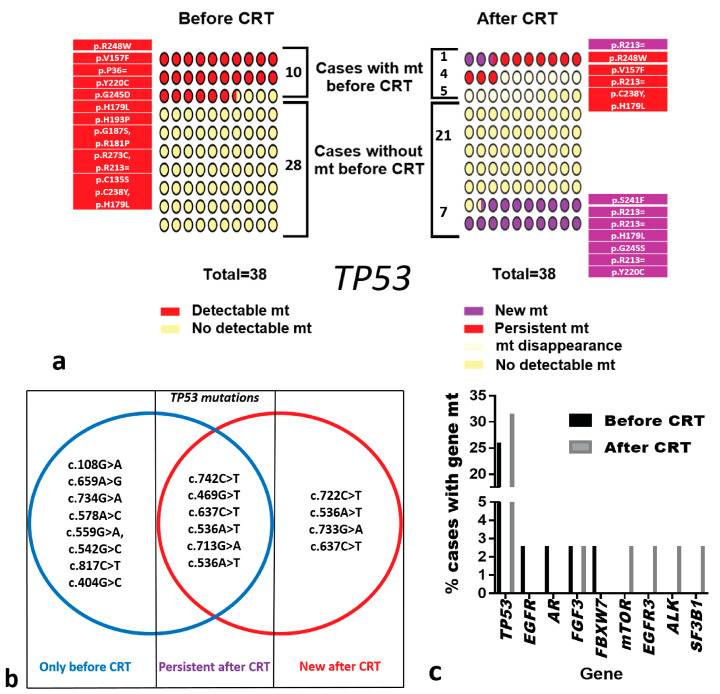
Mutations detected in ctDNA of 38 SCHNC patients: (**a**) *TP53* mutations and % of mutations (changes or new mutations) observed before vs. after CRT. Numbers refer to the actual number of patients, while dots reflect the estimated percentages (per 100 patients). (**b**) Changes that have occurred in the nucleotide sequence in *TP53* (mutations before CRT are included in the blue circle, while mutations after CRT are included in the red circle; the intersection of the two circles includes persistent mutations). (**c**) Percentage of patients with mutations of specific genes detected in ctDNA of 38 SCHNC patients before and after CRT. Abbreviations: CRT = chemo-radiotherapy, mt = mutations.

The baseline pre-CRT analysis showed a clear prevalence of mutations of the *TP53*, recorded in 10/38 (26.3%) of patients. Single mutation was recorded in 7/10 patients, while multiple mutations of the gene were recorded in 3/10 patients (two mutations in two patients and three in one). After CRT, *TP53* mutations were undetectable in 5/10 of these patients, while baseline mutations persisted in 4/10. In one (1/10) additional patient, disappearance of the pre-existing *TP53* mutation was noted, while a new mutation became detectable. Reduction in the number of TP53 mutations was observed in patients who presented with multiple mutations at baseline: patient no. 22 had two mutations post CRT (at baseline, three mutations), and patient no. 35, with two mutations at baseline, had only one post CRT. Both mutations in patient no. 30 were undetectable post CRT. Of interest, mutations of the *TP53* gene were further detected post CRT in 7/38 additional patients with undetectable mutations at baseline. In this way, the rate of *TP53* mutation detection (before and/or after CRT) was 17/38 (44.7%). Figure 1a shows the mutation rates of *TP53* and changes after CRT, while Figure 1b shows the mutations of TP53 before, after CRT, and these persisted throughout therapy.

Analysis of *TP53* mutations detected after CRT according to the chemotherapy regimen applied (cisplatin alone vs. cetuximab with or without cisplatin) did not reveal any statistically significant difference (mutation rate: 4/13 vs. 8/25 patients, respectively; *p* = 0.99). 

Furthermore, 4/38 more patients exhibited mutations of *EGFR*, *AR*, *FGFR3*, and *FBXW3* (one patient, respectively) before CRT. After CRT, the *FGFR3* mutation remained detectable in the patient, while the rest were undetectable. Moreover, four additional mutations became detectable after CRT in four patients (one each) without pre-treatment detectable mutations. These concerned the *MTOR*, *EGFR3*, *ALK*, and *SF3B1* genes, respectively (Table 2). We did not detect any CNVs or gene fusions in the current cohort of patients. Figure 1c shows the genes and mutation rates recorded before and after CRT. Appendix A reports the detected genes with mutations and their main biological functions in cancer biology.

### 3.2. Associations with Histopathological Variables and Patient Age

Supplemental Table S2 reports the distribution of overall and *TP53* mutations according to the age of patients and histopathological variables. For cases with the T4 stage, there was a significantly higher chance of detecting mutations before and after CRT (6/17 T4 patients vs. 1/21 T0-3 patients; *p* = 0.03). No other association with age, T stage, N stage, or histopathological grade was noted. Regarding TP53 mutations, these prevailed in the T4 stage compared to other stages, reaching a maximum significance for patients who had mutations both before and after CRT (5/17 T4 patients vs. 0/21 T0-3 patients; *p* = 0.007).

### 3.3. Associations with Response to CRT

Supplemental Table S3 reports the distribution of overall and *TP53* mutations (recorded before and after CRT) in patients according to the response obtained after CRT. The only statistically significant association concerned the analysis of overall mutations assessed after the end of CRT. Mutations were observed in 8/30 (26.6%) responders (CR/PR) vs. in 6/8 (75%) of the rest of patients (*p* = 0.03). This difference showed a statistical trend after analysis for *TP53* mutations (*p* = 0.08).

### 3.4. Survival Aanalysis

**Figure 2:**
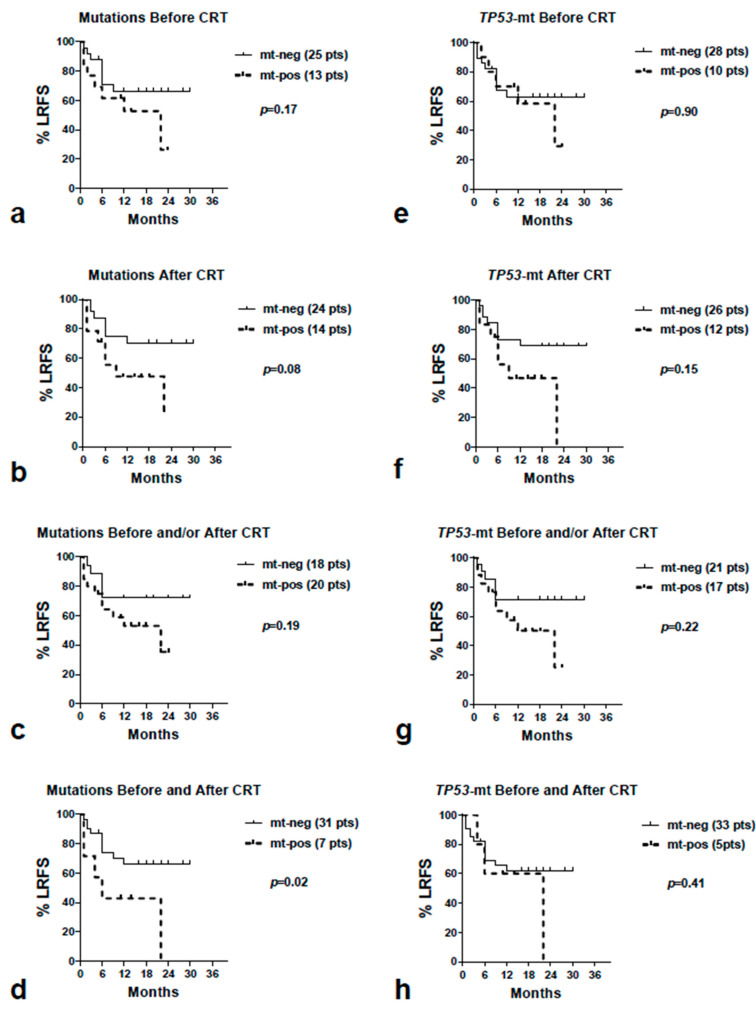
Kaplan–Meier locoregional relapse-free survival curves according to the existence of overall mutations (**a**–**d**) and *TP53* mutations (**e**–**h**) detected before CRT (**a**,**e**), after CRT (**b**,**f**), before and/or after CRT and (**c**,**g**), finally, before and after CRT (**d**,**h**). Abbreviations: CRT = chemo-radiotherapy, LRFS = locoregional relapse-free survival, mt = mutations, neg = negative, pos = positive.

Table 2 and Figure 2 report the univariate and Kaplan–Meier LRFS analysis, according to the presence of overall and *TP53* mutations. Significantly poorer LRFS was noted for patients with persistent detection of mutations (mutations detected before and after CRT) (*p* = 0.02). A marginal association was observed for patients with detectable mutations after CRT (*p* = 0.08).

Univariate tables from Kaplan–Meier loco-regional relapse-free survival, disease-specific overall survival and distant metastasis-free survival analysis according to the presence of overall and *TP53* mutations. The grouping of cases was performed using four variables: i. mutations detected before (B) CRT, ii. mutations detected after (A) CRT, iii. mutations detected before and/or after CRT, and iv. mutations detected before and after CRT. 

**Figure 3:**
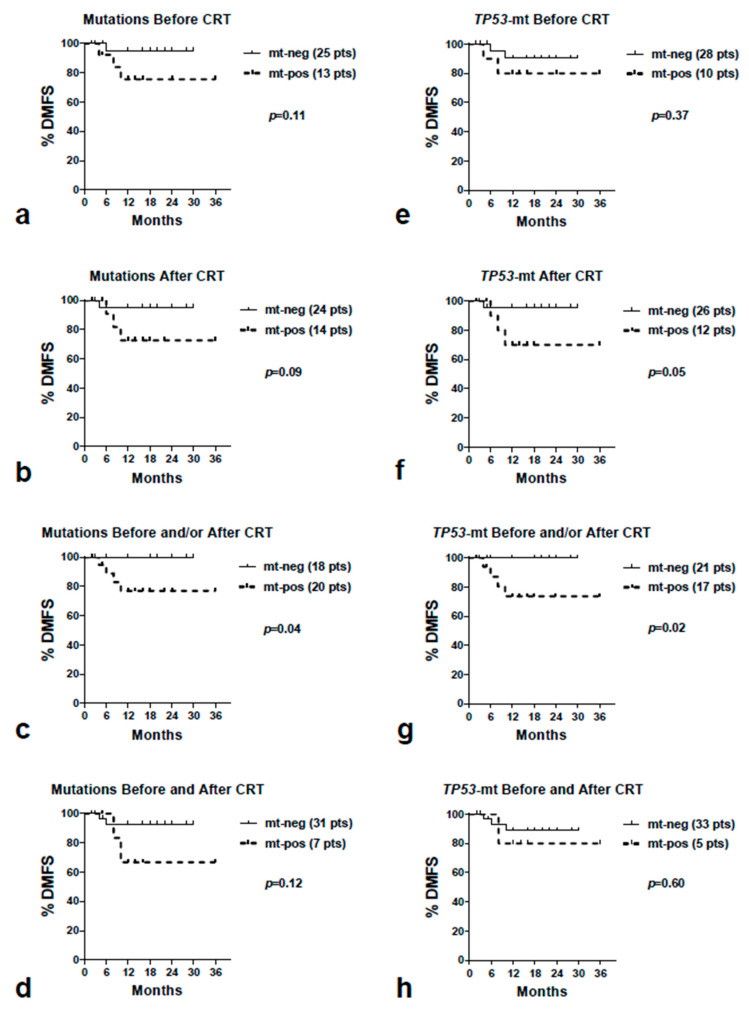
Kaplan–Meier distant metastasis-free survival curves according to the existence of overall mutations (**a**–**d**) and *TP53* mutations (**e**–**h**) detected before CRT (**a**,**e**), after CRT (**b**,**f**), before and/or after CRT (**c**,**g**) and, finally, before and after CRT (**d**,**h**). Abbreviations: DMFS = distant metastasis-free survival, CRT = chemo-radiotherapy, mt = mutations, neg = negative, pos = positive.

We found no association of mutations with the OS (Supplemental Figure S1). Analysis of DMFS showed that patients with mutations after CRT and patients with mutations after CRT had a marginally poorer outcome (*p* = 0.11 and 0.09, respectively), which reached significance for patients who had detectable mutations either before or after CRT (*p* = 0.04) (Table 2 and Figure 3). None of the patients without mutations (before or after CRT) developed metastasis during their follow-up. Analysis of *TP53* mutations showed that patients with mutation after CRT and patients with mutations either before or after CRT had a significant association with poor prognosis (*p* = 0.05 and 0.02, respectively).

### 3.5. Specific Gene Mutations and Disease Progression

Supplemental Tables S4 and S5 present the specific gene mutations recorded before and after CRT, respectively, in patients whose disease progressed or did not progress after CRT. Regarding *TP53* mutations detected before CRT, p.R248W, p.V157F, p.Y220C, p.C238Y and p.C135S characterized patients who progressed after therapy. Regarding *TP53* mutations detected after CRT, p.H179L, p.R213=, p.R248W, p.C238Y and of two newly detected mutations p.S241F, and p.V157F characterized patients who progressed after therapy. 

Among other gene mutations, the p.R505C mutation of the *FBXW7*, the p.E894K mutation of the *AR*, and the p.F384L mutation of the *FGFR3* genes detected before CRT were recorded in patients with disease progression. In addition, detection of the p.F384L mutation of the *FGFR3* and the p.R2217W mutation of the *mTOR* after CRT were found in two patients, respectively, with disease progression.

## 4. Discussion

Gene mutations are frequently present in SCHNCs. These mutations concern genes involved in cell proliferation, survival, and death regulation pathways (e.g., p53 and EGFR signaling pathway), cellular differentiation (e.g., Wnt, NOTCH1, Hedgehog pathway), or regulation of the cell cycle (e.g., cyclins and related genes). *TP53* mutations seem to have a dominant role in the biology of SCHNC [19]. Huang et al. reported that mutations of the *TP53* were noted in tissue samples of 55% of SCHNCs, and this rate was similar in HPV-positive and -negative tumors [20]. Using NGS, TP53 mutations can also be detected in the plasma of SCHNC patients [21]. Economopoulou et al. reported a 32.6% rate of *TP53* mutations in a series of 45 SCHNC patients [22]. Furthermore, in an investigation detecting gene alterations in the ctDNA from the saliva of SCHNC patients, mutations were recorded in 76% of cases [23]. In the current study, we confirmed an evident prevalence of mutations of *TP53* in SCHNCs, which concerned 26.3% of patients examined at baseline. In addition, we identified multiple *TP53* mutations in a minority of patients, while mutations of other genes, like *EGFR, AR, FGFR3,* and *FBXW3*, were noted in 10% of patients. The rate of *TP53* mutations reported herein are similar to the one reported by Economopoulou et al. [22], but certainly lower than the 50–80% rates reported in studies on tissue samples [19,20]. Of interest, Porter et al. and Galot et al. recorded ctDNA *TP53* mutations in 68% and 50% of patients with head and neck cancer, respectively [24,25]. However, in both studies, blood samples were drawn from patients with recurrent or metastatic disease, a parameter that could potentially explain the higher rates of *TP53* mutations. 

In this investigation, we also performed an analysis of gene mutations detected in the blood of SCHNC patients immediately after the end of CRT. In this way, we could identify the persistence, disappearance, or new mutations of genes after therapy. After CRT completion, *TP53* mutations were undetectable in about half of patients with baseline detectable mutations. This may be a result of high intrinsic radiosensitivity and early elimination of cancer cell clones bearing these mutations during the course of CRT. Although *TP53* mutations are involved in apoptosis inhibition and resistance to RT, this effect is not consistent as specific *TP53* mutations have been linked with enhanced apoptotic tendency after irradiation [26]. Additional molecular pathways may also counteract p53-mediated radioresistance and sustain radiosensitivity [27]. For example, the *FBXW7* gene, mutations of which were noted in one patient before CRT, has been shown to confer survival of cancer cells during RT by induction of p53 protein degradation and blockage of apoptosis [28]. 

Persistent detection of specific *TP53* mutations, and, for one case, mutations of *FGFR3*, was noted in about 10% of patients after the end of CRT in our study. In addition, in 18% of the patients with undetectable *TP53* mutations at baseline, new TP53 mutations could be detected post CRT. In this way, the total rate of *TP53* mutations recorded in ctDNA was 44.7%. Moreover, new mutations of other genes, undetectable at baseline, were also recorded in a minority of patients, and these concerned *MTOR, EGFR3, ALK*, and *SF3B1*. Emerging mutations in patients with esophageal cancer progressing after CRT have also been noticed in a study by Azad et al. [29]. Persistence of baseline mutations and the emergence of new detectable mutations could indicate radioresistance of the cancer cell compartment bearing these very gene mutations. Indeed, in the current study, patients with detectable mutations after CRT had a significantly lower tumor response rate. In this context, an interesting study in medulloblastoma suggested that the dominant clone at recurrence after RT emerges through selections of pre-existing minor clones [30], which may also apply to patients where new mutations were recorded after CRT.

As far as prognosis is concerned, patients with persistent detection of mutations after CRT (detectable mutations at baseline) had significantly worse LRFS. A marginal association was also noted for patients with mutations detected after CRT. Although we found no significant association with OS, patients with detectable mutations after CRT, or mutations before and/or after CRT had a significantly higher rate of development of distant metastases. This finding was noted after taking into account all gene mutations and when analysis concerned *TP53* mutations only. A retrospective mutation analysis of the ctDNA of 75 patients with SCHNC (stages I–IV, stage IV 62.7%) demonstrated that both overall ctDNA alterations and *TP53* mutations significantly correlated with advanced tumor progression status and OS [31]. In addition, it has been reported that the presence of ctDNA mutations either before or before and after treatment with CRT was linked with decreased survival [22]. Taylor et al. published the results of a study in SCHNC patients treated with chemotherapy or immunotherapy, suggesting that, although baseline ctDNA abundance was not associated with OS, changes in the ctDNA variant allele frequency were predictive of progression-free survival [32]. Two additional studies, although performed in squamous cell esophageal cancer, showed a significant association of ctDNA mutations with prognosis. Wang et al. reported that detectable ctDNA alterations one or several months after RT were linked with inferior progression-free survival of patients, while a better prognosis was recorded for patients whose ctDNA disappeared one month after therapy [33]. Azad et al. also found an increased risk of disease progression in patients with squamous cell esophageal cancer when ctDNA mutations were recorded after CRT [29].

Beyond the well-known limitations related to the NGS procedure (quality of the isolated DNA, bioinformatic analysis variations, false negative results), other limitations of the study include the relatively low number of patients recruited in the prospective trial due to predefined funding and the high cost of NGS experiments. Moreover, although the study focused on SCHNC, this includes different primary tumor locations with eventual different pathogenesis, clinical behavior and prognosis. In addition, the HPV status was not studied in parallel with NGS. Longer follow-up could also have allowed the extraction of more robust conclusions. Nevertheless, the treatment was consistent for all patients and the inclusion of liquid biopsies after the end of therapy provided further insights of the biology behind the interplay of CRT with tumor biology. 

## 5. Conclusions

Despite the aforementioned limitations, it is suggested that detection of *TP53* and other gene mutations in the ctDNA from the plasma of patients with SCHNC treated with radical CRT can be achieved with NGS. Assessment of mutations before and after the end of CRT is, however, essential to characterize patients with high risk of locoregional recurrence or even metastatic progression. Persistent detection of mutations, pre-existing or new, appeared as the major identified parameter that predicted locoregional progression after CRT. Although *TP53* mutations prevailed, detection of less frequently recorded mutations of other genes, like *FGFR3, MTOR*, *EGFR3*, *ALK*, and *SF3B1,* mutations after CRT seem also to contribute to the overall association of the mutational burden with disease progression. The genomic alterations post CRT described herein provide a platform for novel therapeutic approaches for SCHNC that test combined targeted therapies and CRT.

## Figures and Tables

**Table 1:** Gene mutations detected in ctDNA before and after chemo-radiotherapy.

	Before CRT				After CRT			
No. Patient	Gene (No)	AA Mutation	CDS Mutation	Molecular Frequency %	Gene (No)	AA Mutation	CDS Mutation	Molecular Frequency %
	***TP53***				***TP53***			
1	1	p.R248W	c.742C>T	1.2	1	p.R248W	c.742C>T	0.49
11	1	p.V157F	c.469G>T	54	1	p.V157F	c.469G>T	126
13	1	p.P36=	c.108G>A	52	0	-----	----	-----
17	1	p.Y220C	c.659A>G	1.18	0	-----	----	-----
18	1	p.G245D	c.734G>A	1.68	1	p.R213=	c.637C>T	52
29	1	p.H179L	c.536A>T	0.42	0	-----	----	-----
38	1	p.H193P	c.578A>C	2.14	0	-----	----	-----
30	2	p.G187S p.R181P	c.559G>A, c.542G>C	1.60 2.40	0	----- -----	----- -----	----- -----
35	2	p.R273C p.R213=	c.817C>T c.637C>T	0.17 0.32	1	----- p.R213=	----- c.637C>T	0.48
22	3	p.C135S p.C238Y p.H179L	c.404G>C c.713G>A c.536A>T	1.34 0.39 41	2	----- p.C238Y p.H179L	----- c.713G>A c.536A>T	----- 0.34 3.70
10	0	-----	----	----	1	p.S241F	c.722C>T	15
20	0	-----	----	----	1	p.R213=	c.637C>T	1.08
23	0	-----	----	----	1	p.R213=	c.637C>T	51
25	0	-----	----	----		p.H179L	c.536A>T	0.39
26	0	-----	----	----	1	p.G245S	c.733G>A	0.27
27	0	-----	----	----	1	p.R213=	c.637C>T	0.27
33	0	-----	----	----	1	p.Y220C	c.659A>G	1.17
	***EGFR***				***EGFR***			
28	1	p.P848L	c.2543C>T	0.10	0	-----	----	----
	***AR***				***AR***			
10	1	p.E894K	c.2680G>A	0.49	0	-----	----	----
	***FGFR3***				***FGFR3***			
16	1	p.F384L	c.1150T>C	48	1	p.F384L	c.1150T>C	46
	***FBXW7***				***FBXW7***			
10	1	p.R505C	c.1513C>T	269	0	----	----	----
	***mTOR***				***mTOR***			
23	0	----	----	----	1	p.R2217W	unknown	0.24
	***EGFR3***				***EGFR3***			
27	0	----	----	----	1	p.V104M	c.310G>A	0.16
	***ALK***				***ALK***			
26	0	----	----	----	1	p.R1275Q	c.382G>A	0.17
	***SF3B1***				***SF3B1***			
9	0	-----	----	----	1	p.K700E	c.2098A>G	0.62

**Table 2:** Univariate tables from Kaplan–Meier loco-regional relapse-free survival, disease-specific overall survival and distant metastasis-free survival analysis according to the presence of overall and *TP53* mutations. The grouping of cases was performed using 4 variables: i mutations detected before (B) chemo-radiotherapy (CRT), ii. mutations detected after (A) CRT, iii. mutations detected before and/or after CRT, and iv. mutations detected before and after CRT. Abbreviations: LRFS = locoregional relapse-free survival, OS = disease-specific overall survival, DMFS = distant metastasis-free survival.

	All Mutations								
	LRFS			OS			DMFS		
Yes vs. No	***p*-value**	**HR**	**95%CI**	**No**	**Yes**	***p*-value**	**No**	**Yes**	*p*-value (*)
B	0.17	2.20	0.7–6.8	0.93	1.05	0.3–3.6	0.11	5.12	0.6–39
A	0.08	3.10	0.9–9.6	0.51	1.52	0.4–5.2	0.09	5.75	0.7–44
B and/or A	0.19	2.28	0.8–6.0	0.43	1.60	0.4–5.2	0.04	6.75	0.9–48
B and A	0.02	5.38	1.2–24	0.93	0.93	0.2–4.2	0.12	6.82	0.5–80
Yes vs. No	***TP53* mutations**								
B	0.90	1.42	0.4–1.6	0.98	0.98	024–3.7	0.37	2.66	0.3–22
A	0.15	3.01	0.9–9.9	0.71	1.27	0.3–4.0	0.05	7.75	0.9–64
B and/or A	0.22	2.42	0.8–7.0	0.49	1.51	0.4–5.0	0.02	9.19	1.2–65
B and A	0.41	1.90	0.4–8.9	0.61	0.64	0.1–3.6	0.60	2.03	0.1–30

B = before CRT, A = After CRT, No = no detected mutations, Yes = detected mutations.

## Data Availability

All data are available in the files of the Department of Radiotherapy. and Oncology, Democritus University of Thrace. The data presented in this study are available on reasonable request from the corresponding author.

## Supplementary Materials

The following supporting information can be downloaded at: https://www.mdpi.com/article/10.3390/curroncol30100643/s1, Figure S1: Kaplan-Meier disease specific overall survival curves according to the existence of overall mutations and *TP53* mutations detected before chemo-radiotherapy, after CRT, before and/or after CRT and, finally, before and after CRT; Table S1: Patient and disease characteristics; Table S2: Distribution of overall and *TP53* mutations, according to the age of patients and histopathological variables; Table S3: Distribution of overall and *TP53* mutations; Table S4: Distribution of specific gene mutations before CRT in patients according to the progression status (after CRT); Table S5: Distribution of specific gene mutations at the end of CRT in patients according to the progression status (after CRT).

## Appendix A

**Table A1:** Mutated genes and principal functions.

GENE	FUNCTION
*TP53*	A tumor suppressor gene. Encodes the tumor protein p53, a crucial regulator of apoptotic response, and guardian of the genome integrity. It also regulates DNA repair proteins and can induce cell cycle arrest at the G1/S cell cycle phase. Also involved in cellular senescence.
*EGFR/ErbB1*	The Epidermal Growth Factor Receptor or *ErbB-1* gene encodes a transmembrane receptor that is activated by specific ligands like EGF and TGF-α, initiating a cascade of signaling events involved in proliferation, metabolism and resistance to chemotherapy and radiotherapy. Amplification and mutations of the gene promote aberrant activation lading to carcinogenesis and tumor progression.
*AR*	Androgen receptor gene encodes ARs, transcription factors that, following their binding to testosterone, enter the nuclei to activate several genes involved in tumor progression.
*FGFR3*	Encodes a member of the fibroblast growth factor receptor family, a membrane protein that binds to the fibroblast growth factors of the tumor stroma, promoting proliferation and differentiation. Mutations of the FGFR3 have been detected in bladder cancer and glioblastomas and are involved in cell proliferation and resistance to anti-cancer therapy.
*FBXW7*	The F-box and WD repeat domain containing 7 gene encodes a member of the F-box protein family with critical tumor suppressor functions. It controls the degradation of several oncoproteins (c-myc, mcl-2, mTOR, jun, cycline E) through the proteasome pathway. Its mutations promote carcinogenesis and tumor growth.
*mTOR*	The mammalian target of rapamycin gene regulates cell proliferation, autophagy, apoptosis and metabolism pathways including glycolysis. Its mutations promote carcinogenesis.
*ErbB3*	Encodes a member of the EGFR family protein. Activating mutations lead to resistance to anti-cancer therapy.
*ALK*	The anaplastic lymphoma kinase gene can be activated in a subgroup of solid tumors, driving cell growth and resistance to chemotherapy. Specific targeting drugs have been approved for the treatment of ALK-positive patients with lung cancer.
*SF3B1*	It encodes subunit 1 of the splicing factor 3b protein complex. Mutations of the gene are linked with chronic lymphocytic leukemia, myelodysplastic syndromes, breast cancer, and orbital melanoma.
