# Supplementary material for: Next-Generation Sequencing Analysis of Mutations in Circulating Tumor DNA from the Plasma of Patients with Head–Neck Cancer Undergoing Chemo-Radiotherapy Using a Pan-Cancer Cell-Free Assay
Source: Curr Oncol. 2023 Sep 29;30(10):8902–15. doi: 10.3390/curroncol30100643 (PMC10604986; doi:10.3390/curroncol30100643)
Supplement: Supplementary file 1 [file curroncol-30-00643-s001.zip › curroncol-2592993-supplementary.pdf]

Table S1. Patient and disease characteristics. .

No Patients	38
Age	
range	32-83
Median	64
Performance Status	
Median	0-1
Range	0
Sex	
Male	34
Female	4
Location	
Larynx	17
Oropharynx	3
Oral cavity	5
Hypopharynx	3
Nasopharynx	6
Parotid gland	2
Neck	2
Histology (*)	
Squamous grade 1	6
Squamous grade 2	22
Squamous grade 3	10
T-stage	
T0(**)	2
T2	5
T3	14
T4	17
N-stage	
N0	14
N1	6
N2	7
N3	11

(*) conventional type of squamous cell head-neck cancer

(**) nodal disease with no detectable primary tumor

Table S2. Distribution of overall and *TP53* mutations, according to the age of patients and histopathological variable. The grouping of cases was performed using 4 variables: 1. mutations detected before chemo-radiotherapy (CRT), ii. mutations detected after CRT, iii. mutations detected before and/or after CRT, and iv. mutations detected before and after CRT.

	Mutations					*TP53* mutations			
Parameter	Before CRT	After CRT	B and/or A CRT	B and A CRT		Before CRT	After CRT	B and/or A CRT	B and A CRT
	No Yes p-value	No Yes p-value	No Yes p-value	No Yes p-value		No Yes p-value	No Yes p-value	No Yes p-value	No Yes p-value
No pts	25 13	24 14	18 20	31 7		28 10	26 12	21 17	33 5
Age<br><60<br>>60	 11 6 0.99<br>14 7	 13 4 0.12<br>11 10	 9 8 0.53<br>9 12	 15 2 0.34<br>16 5		 12 5 0.72<br>16 5	 11 6 0.65<br>15 6	 10 7 0.69<br>11 10	 14 3 0.73<br>19 2
Location<br>Larynx<br>Oropharynx<br>Oral cavity<br>Hypopharynx<br>Nasopharynx<br>Parotid<br>Neck	 10 7 0.20<br>3 0<br>3 2<br>3 0<br>2 4<br>2 0<br>2 0	 9 8 0.17<br>3 0<br>3 2<br>3 0<br>5 1<br>0 2<br>1 1	 7 10 0.15<br>3 0<br>2 3<br>3 0<br>2 4<br>0 2<br>1 1	 12 5 0.72<br>3 0<br>4 1<br>3 0<br>5 1<br>2 0<br>2 0		 11 6 0.17<br>3 0<br>4 1<br>3 0<br>3 3<br>2 0<br>2 0	 9 8 0.09<br>3 0<br>4 1<br>3 0<br>5 1<br>0 2<br>2 0	 7 10 0.39<br>3 0<br>3 2<br>3 2<br>3 3<br>0 2<br>2 1	 13 4 0.68<br>3 0<br>5 0<br>3 0<br>5 1<br>2 0<br>2 0
T-stage (*)<br>T0<br>T2<br>T3<br>T4	 2 0 0.17<br>3 2<br>11 3<br>9 8	 1 1 0.09<br>4 1<br>11 3<br>8 9	 1 1 0.20<br>3 2<br>8 6<br>6 11	 2 0 0.03<br>4 1<br>14 0<br>11 6		 2 0 0.07<br>4 1<br>12 2<br>10 7	 2 0 0.01<br>5 0<br>11 3<br>8 9	 1 0 0.02<br>4 1<br>9 5<br>6 11	 2 0 0.007<br>5 0<br>14 0<br>12 5
N-stage (**)<br>N0<br>N1<br>N2<br>N3	 9 5 0.47<br>4 2<br>5 2<br>8 3	 10 4 0.80<br>3 3<br>6 1<br>5 6	 8 6 0.72<br>2 4<br>4 3<br>4 7	 11 3 0.75<br>5 1<br>7 0<br>8 3		 11 3 0.84<br>4 2<br>6 1<br>7 4	 11 3 0.82<br>3 3<br>6 1<br>6 5	 9 5 0.97<br>2 4<br>5 2<br>5 6	 13 1 0.83<br>5 1<br>7 0<br>8 3
Grade (***)<br>1<br>2<br>3	 4 2 0.20<br>15 7<br>6 4	 5 1 0.53<br>13 9<br>6 4	 3 3 0.86<br>11 11<br>4 6	 6 0 0.45<br>17 5<br>8 2		 4 2 0.83<br>17 5<br>7 3	 5 1 0.62<br>15 7<br>6 4	 3 3 0.44<br>14 8<br>4 6	 6 0 0.47<br>18 4<br>9 1

(*) T0,2,3 vs. T4; (**) N0,1 vs. N2,3 ; (***) chi-square – all parameters

Table S3. Distribution of overall and *TP53* mutations (No= undetected mutations vs. Yes= detected mutations) according to the response obtained with CRT. The grouping of cases was performed using 4 variables: i. mutations detected before (B) chemo-radiotherapy (CRT), ii. mutations detected after (A) CRT, iii. mutations detected before and/or after CRT, and iv. mutations detected before and after CRT.

	**All mutations**			
**Response**	**Before CRT**	**After CRT**	**B and/or A (**)**	**B and A CRT (***)**
	**No Yes p-value(*)**	**No Yes p-value(*)**	**No Yes p-value(*)**	**No Yes p-value(*)**
All cases	*25 13*	*24 14*	*18 20*	*31 7*
CR<br>PR<br>MR/SD<br>PgD	16 8 0.99<br>4 2<br>3 2<br>2 1	18 6 0.03<br>4 2<br>1 4<br>1 2	13 11 0.99<br>3 3<br>1 4<br>1 2	21 3 0.14<br>5 1<br>3 2<br>2 1
	***TP53* mutations**			
All cases	*28 10*	*26 12*	*21 17*	*33 5*
CR<br>PR<br>MR/SD<br>PgD	17 7 0.65<br>4 2<br>4 1<br>3 0	19 5 0.08<br>4 2<br>2 3<br>1 2	15 9 0.42<br>3 3<br>2 3<br>1 2	21 3 0.99<br>5 1<br>4 1<br>3 0

Table S4. Distribution of specific gene mutations (mt) before CRT in patients according to the progression status (after CRT).

No pt	TP53														EGFR	FBXW7	AR	FGFR3
	p.P36=	p.G245D	p.H179L	p.G187S	p.R181P	p.R273C	p.R213=	p.H193P	p.R248W	p.V157F	p.Y220C	p.C238Y	p.C135S		p.P848L	p.R505C	p.E894K	p.F384L
Patients without disease progression after CRT																		
13	mt																	
15																		
18		mt																
28															mt			
29			mt															
30				mt	mt													
35						mt	mt											
37																		
38								mt										
Patients with disease progression after CRT																		
1									mt									
10																mt	mt	
11										mt								
16																		mt
17											mt							
22			mt									mt	mt					

Table S5. Distribution of specific gene mutations (mt) at the end of CRT in patients according to the progression status (after CRT).

	TP53									SF3B1	ALK	ERBB3	FGFR3	mTOR
No pt	p.G245D	p.H179L	p.R213=	p.R248W	p.Y220C	p.C238Y	p.S241F	p.V157F		p.K700E	p.R1275Q	p.V104M	p.F384L	p.R2217W
	Patients without disease progression after CRT													
9										mt				
18			mt											
26	mt										mt			
27			mt									mt		
33					mt									
35			mt											
	Patients with disease progression after CRT													
1				mt										
10							mt							
11								mt						
16													mt	
20			mt											
22		mt				mt								
23			mt											mt
25		mt												

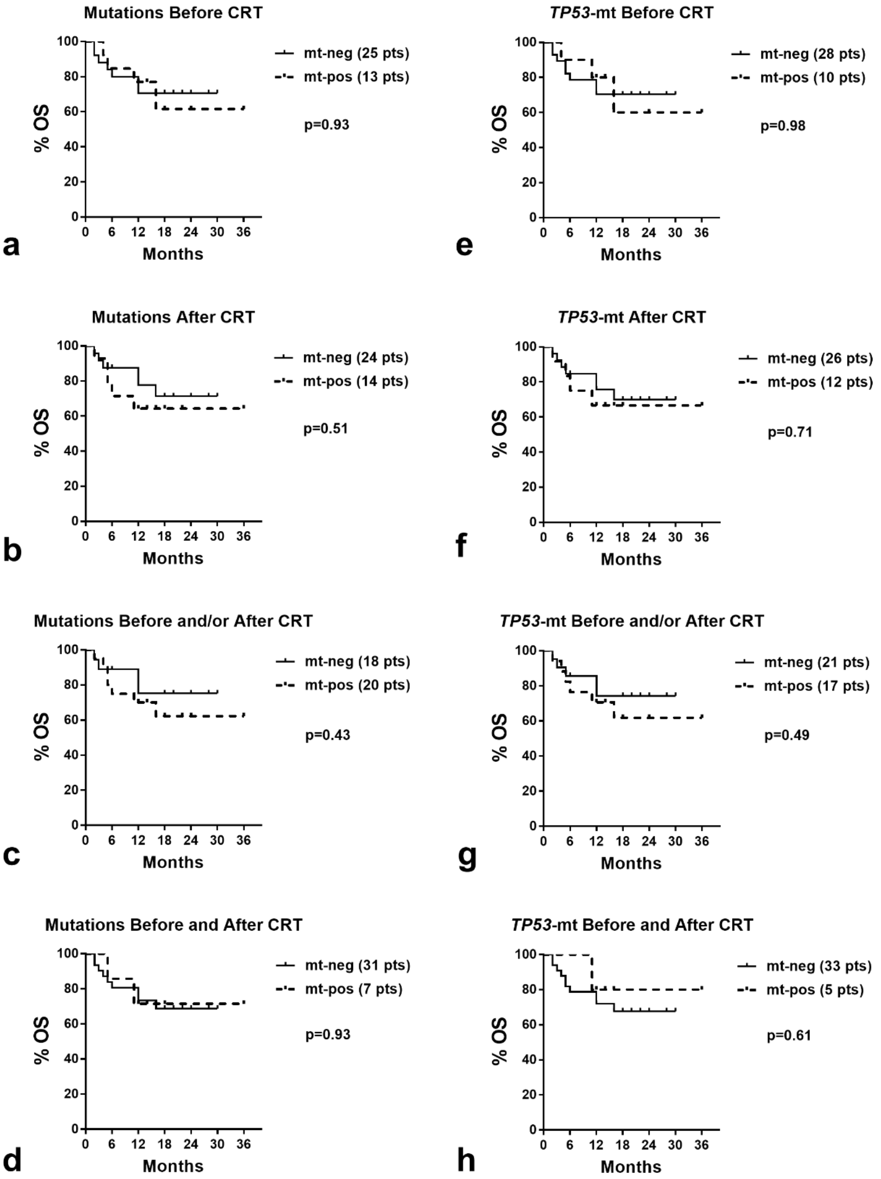

Figure S1. Kaplan-Meier disease specific overall survival curves according to the existence of overall mutations and *TP53* mutations detected before chemo-radiotherapy (CRT), after CRT, before and/or after CRT and, finally, before and after CRT. Abbreviations: OS=disease specific overall survival, CRT=chemo-radiotherapy, mt=mutations, neg=negative, pos=positive.
